# Supplementary material for: High-performance van der Waals antiferroelectric CuCrP2S6-based memristors
Source: Nat Commun. 2023 Nov 30;14:7891. doi: 10.1038/s41467-023-43628-x (PMC10689492; doi:10.1038/s41467-023-43628-x)
Supplement: Supplementary file 1 — Supplementary Information [file 41467_2023_43628_MOESM1_ESM.pdf]

Supplementary Materials

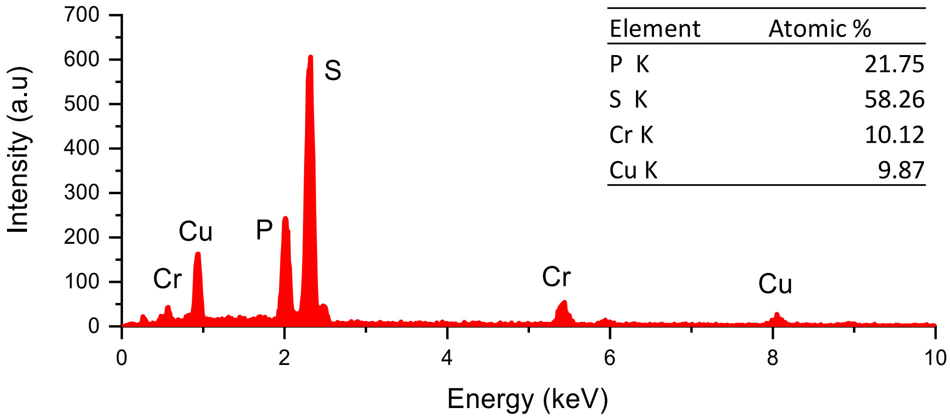

Supplementary Fig. 1 | EDS spectrum of $CuCrP_2S_6$.

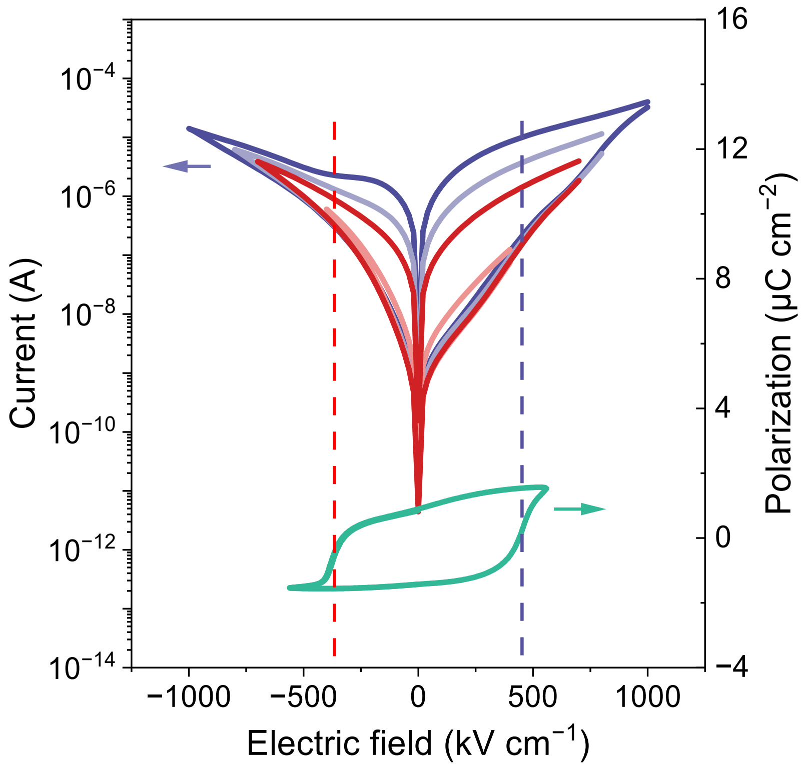

Supplementary Fig. 2 | Comparison of the *I–V* curve and the *P–E* curve regarding electric fields.

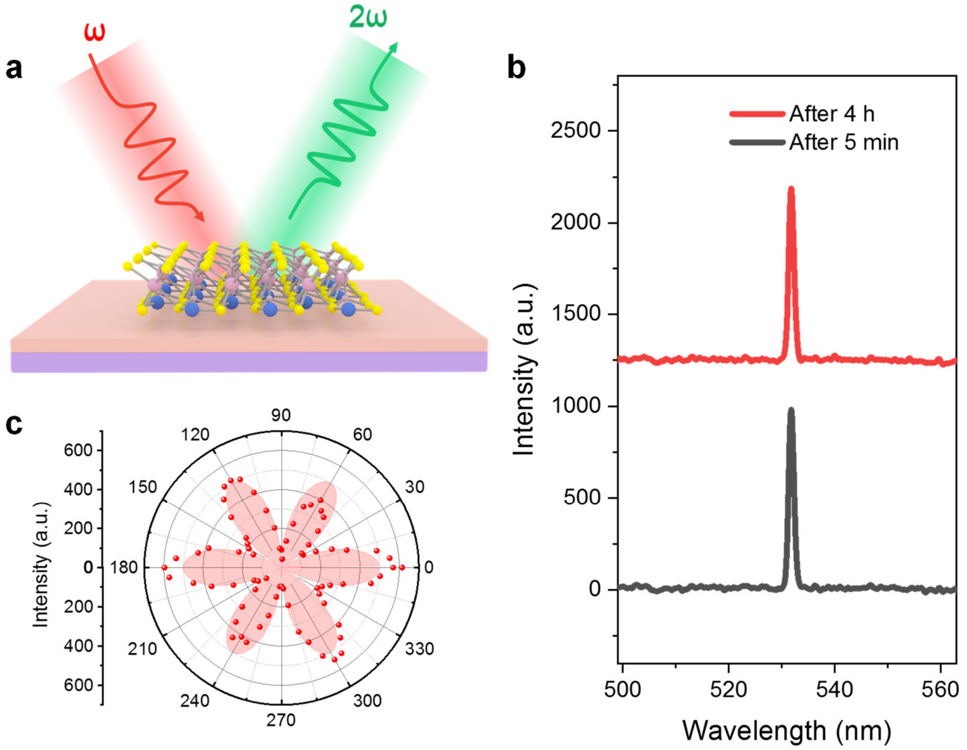

Supplementary Fig. 3 | SHG measurement. a, Schematic of SHG measurement. **b,** SHG peaks on $CuCrP_2S_6$ for 5 minutes and 4 hours later after poling. **c,** Polar plot of SHG intensity for poled $CuCrP_2S_6$ flakes.

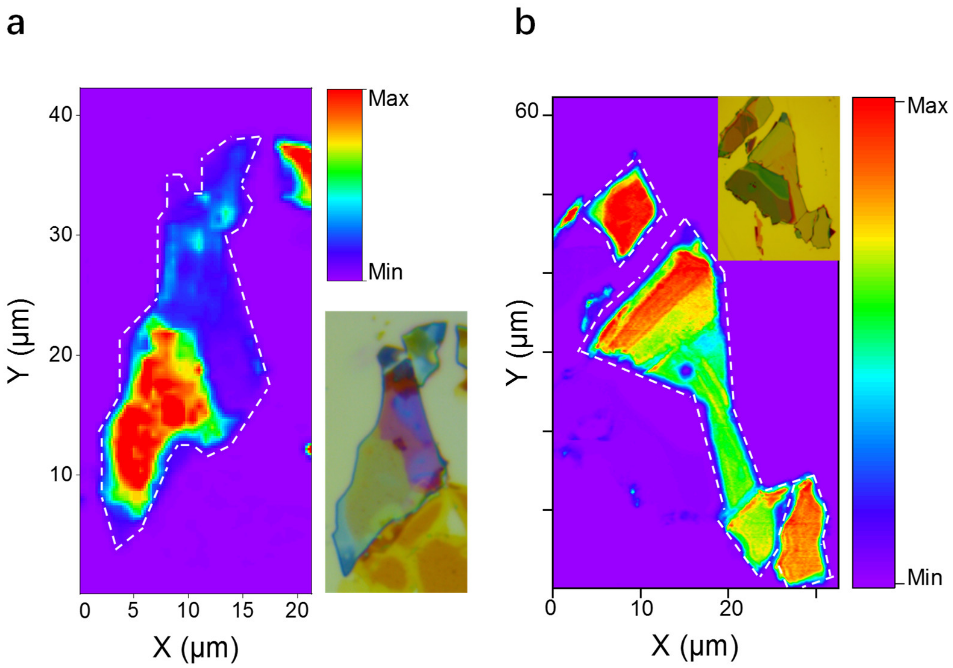

Supplementary Fig. 4 | Mapping of the normalized SHG intensity on poled flakes. Insets: optical images of the flakes. The outlines of poled flakes are marked by dashed lines.

Supplementary Note 1: Probing FE phase using SHG

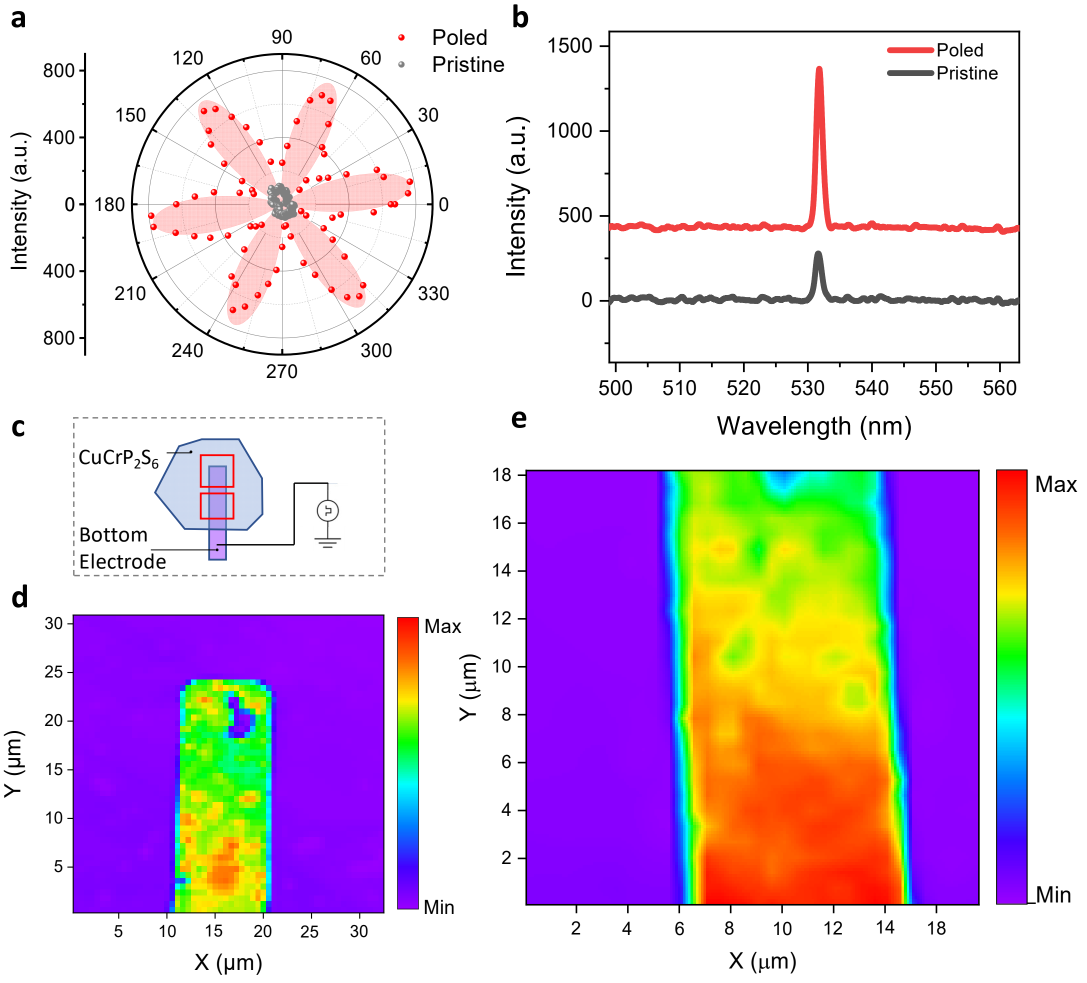

Supplementary Fig. 5 | SHG intensity enhanced by electrical poling. a, Polar plot of SHG intensity for both pristine and poled flakes. **b,** SHG peaks on $CuCrP_2S_6$ at 532 nm for pristine and poled flakes. **c,** The illustration of SHG measurement setup. The $CuCrP_2S_6$ flake was transferred onto the electrode connected to a voltage source. The red boxes mark SHG mapping areas. **d,e,** Normalized SHG intensity mapping for the positions marked in **a**. Laser power: 5 mW.

Under the excitation of a 1064 nm laser pulse, poled $CuCrP_2S_6$ flakes generate a strong SHG signal (Supplementary Fig. 5b), indicating a broken inversion centrosymmetry associated with ferroelectricity, which provides compelling evidence for the presence of ferroelectricity. The electrically poled region exhibits a stronger intensity than the pristine region. The polar plot of the SHG intensity measured for both pristine and poled samples is shown in Supplementary Fig. 5a. The pronounced six-fold symmetry agrees with the hexagonal symmetry viewed along the c-axis direction in the $CuCrP_2S_6$ crystal structure. Supplementary Fig. 5c illustrates the experimental setup where a $CuCrP_2S_6$ flake was transferred onto an electrode

connected to a voltage source. As shown in Supplementary Fig. 5d,e, after the application of 6 V on the bottom electrode, the portion above the electrode produces an increase in SHG intensity. Such a greatly enhanced SHG intensity for the poled crystal reveals the effect of the electric-field induced antiferroelectric-to-ferroelectric transition.

Supplementary Note 2: SKPM measurement

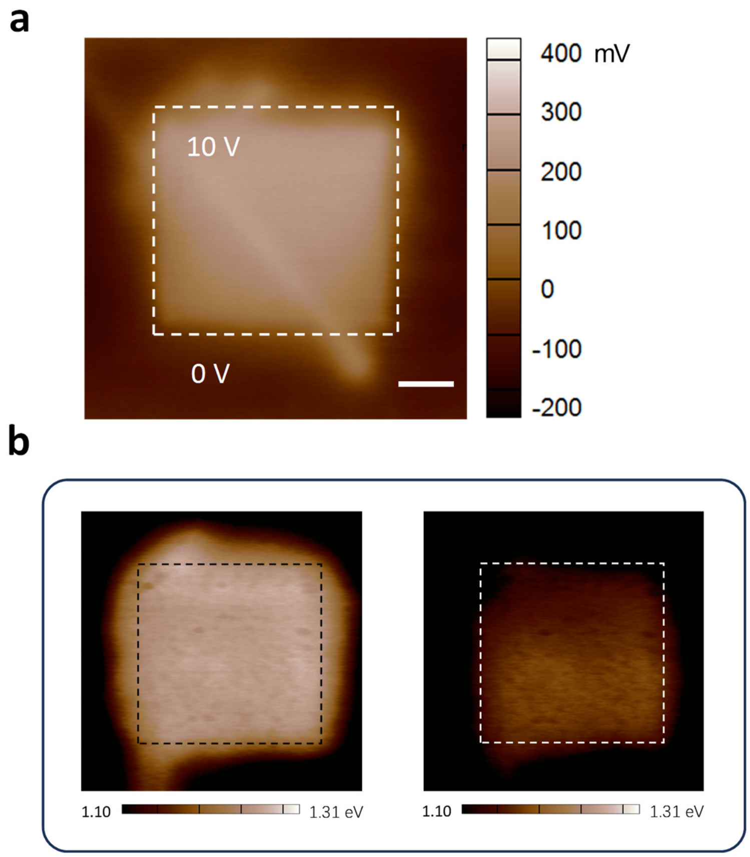

Supplementary Fig. 6 | SKPM measurement. a, Surface potential mapping measured using SKPM. **b,** Retention property for surface potential. Poled regions are marked by dashed lines. Surface potential mapping in the left and right panels were acquired with 5-minute and 1-week delay after poled with the PFM tip, respectively.

There is a direct correlation between polarization states and their surface potentials. Regions with aligned polarization tend to electrostatically induce positive/negative charges over the interfaces, leading to variations in the surface potential. In Supplementary Fig. 6, the local surface potential image for both pristine (outside the square) and poled (inside the square) areas are presented, revealing a clear distinction between them. The surface-potential difference of the modified area is found to be 500 meV higher than that of the unpoled area. This SKPM measurement provides additional information that confirms the PFM results. The poled region exhibits considerably high surface potential, while after one week has significantly relaxed to the ground state. This further substantiates the ferroelectric–antiferroelectric relaxation process.

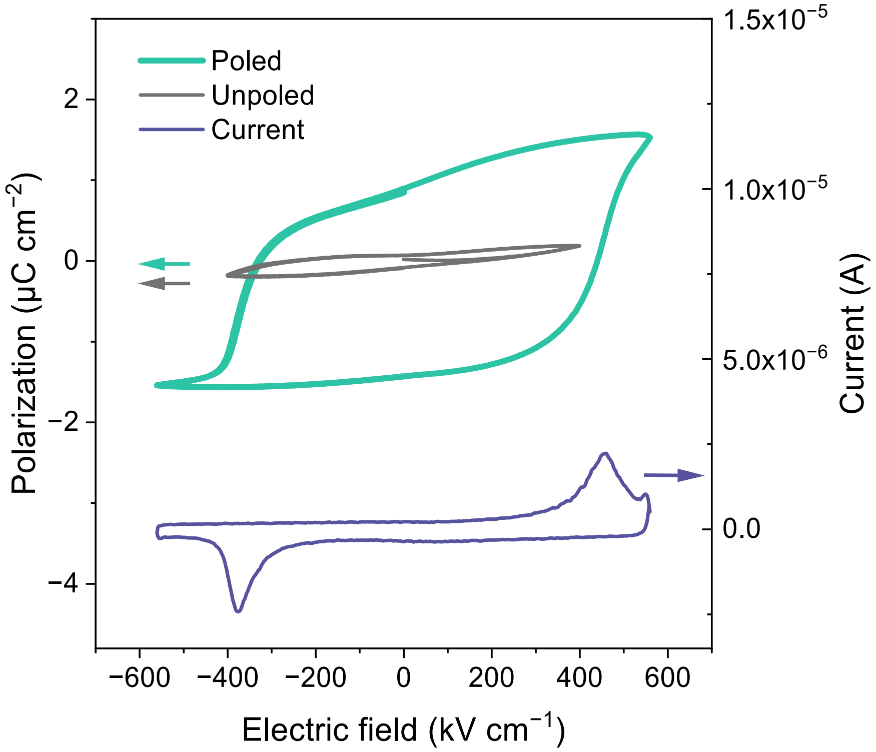

Supplementary Fig. 7 | *P*–*E* hysteresis loops for unpoled and poled samples and the corresponding leakage current measured on the $CuCrP_2S_6$ ferroelectric capacitor after poling. Poling electric field: (600 kV cm^{-1}). Test frequency: 100 Hz.

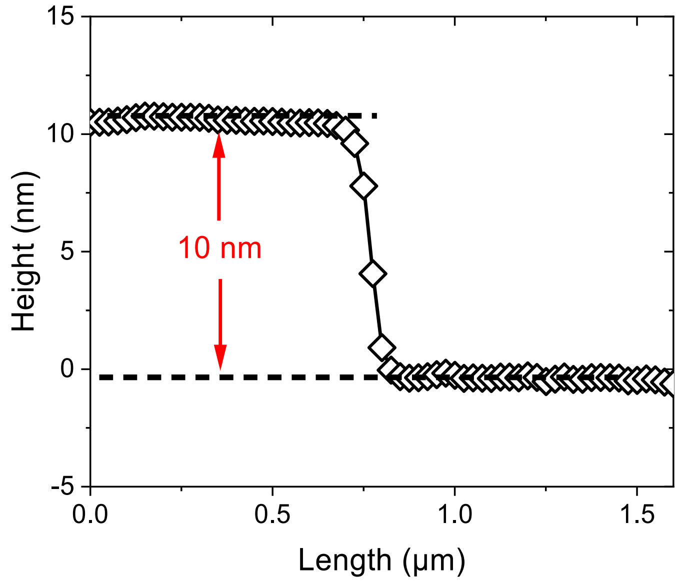

Supplementary Fig. 8 | Height profile along the white dashed line in Fig. 2c measured by AFM.

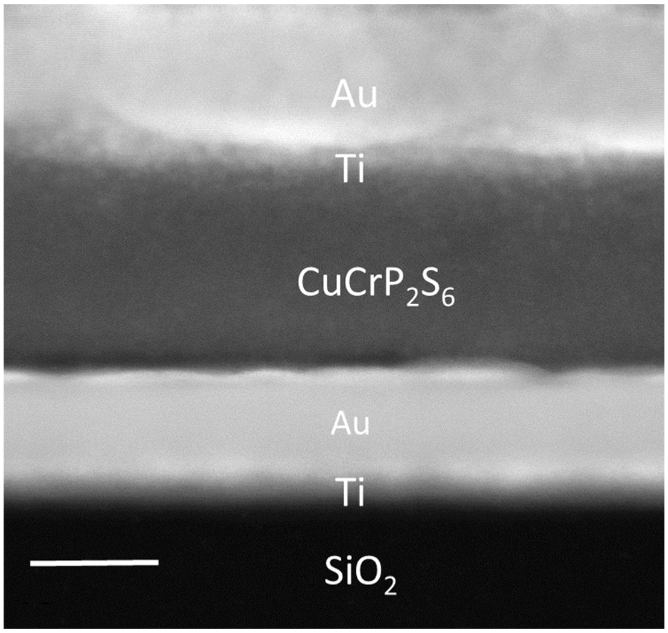

Supplementary Fig. 9 | Cross-sectional TEM image of the memristor. Scale bar: 10 nm.

Supplementary Note 3: Breakdown test

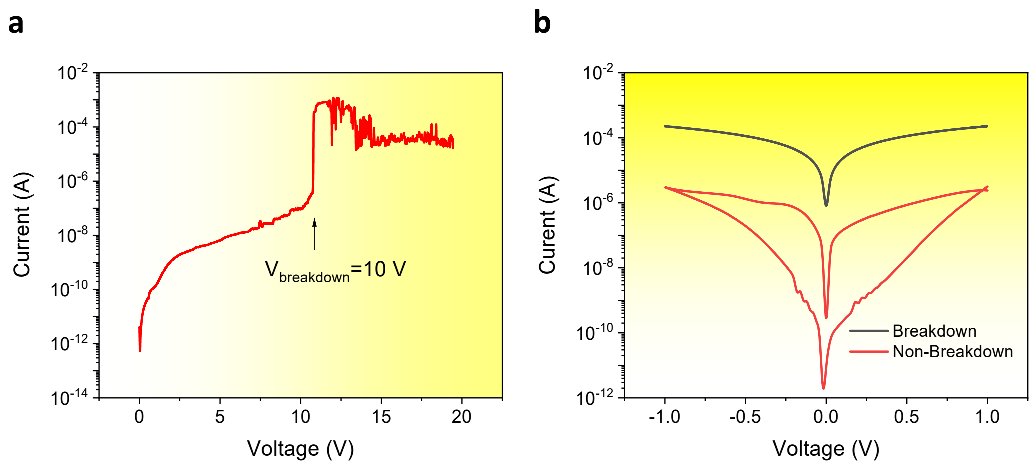

Supplementary Fig. 10 | Device breakdown test. a, Device breakdown at 10 V. **b,** *I–V* characteristics of device before and after breakdown.

Concerning the breakdown of the crystal under a high voltage, we found that the crystal collapses when it suffers from a high voltage beyond the coercive field for a long time. This breakdown is caused by the long-range migration of Cu ions from the octahedral sulfur framework where they originally reside, thus showing a delay effect upon poling the crystal. In our experiment, the breakdown voltage is around 10 V/10 nm (Supplementary Fig. 10), and that breakdown will most likely occur after applying this voltage for more than 5 min. After the breakdown, the device exhibits a short-circuit behavior and loses resistive-switching characteristics (Supplementary Fig. 10b). To avoid the breakdown, the working voltage was limited to 1 V/10 nm and below, and the pulse length was limited to 3 s and below. We ensured that the crystal was not subject to the breakdown condition, allowing for safe and reliable switching operation without causing any permanent damage to the device.

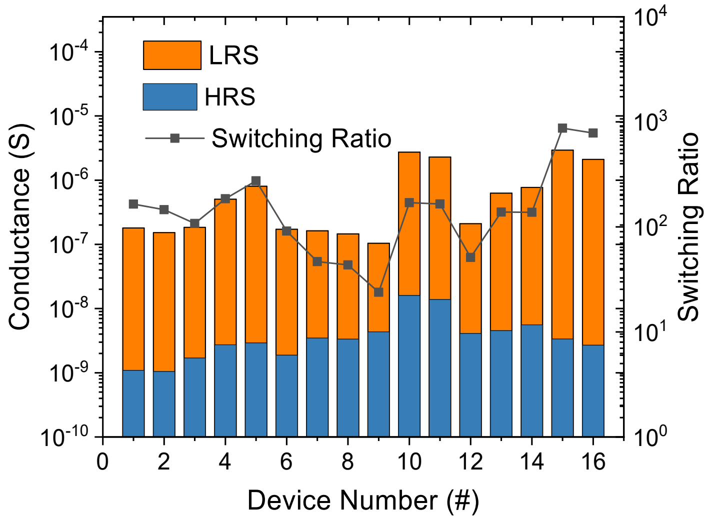

Supplementary Fig. 11 | HRS and LRS conductance and switching ratios of the 16 devices in the 4×4 array.

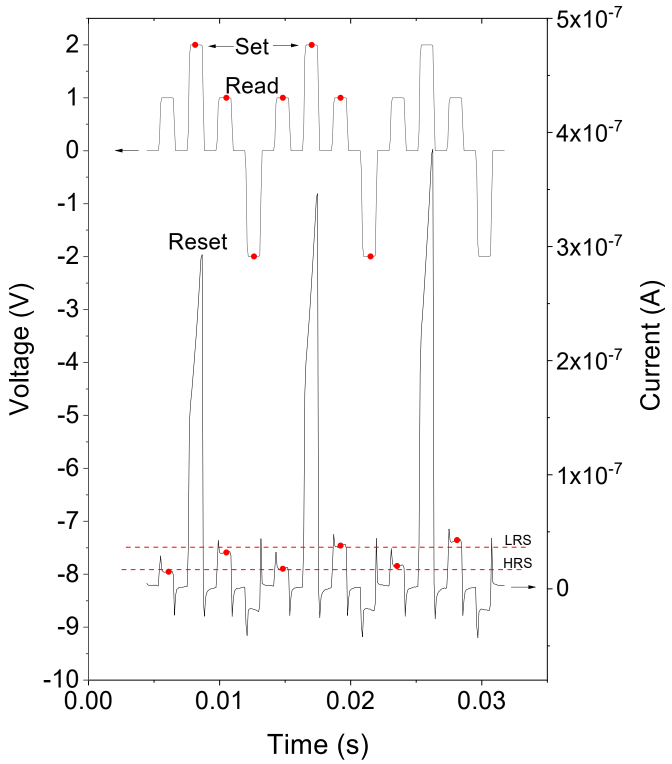

Supplementary Fig. 12 | Voltage waveform and current output of the endurance test for three cycles. The pulses are 2 V and −2 V for the "set" and "reset", respectively. Between the "set" and "reset", there is a pulse of 1 V (1 ms) for reading the resistance. The LRS and HRS are marked by the dashed lines.

Supplementary Note 4: Ion migration-related filamentary switching

High-temperature treated devices rule out the impact of ion-related filamentary switching. It is widely known that ion migration contributes to resistive switching by forming ionic conductive filaments (Supplementary Fig. 13c). Resistive switching tends to exhibit abrupt changes upon filament formation. We found that a minority of devices exhibit such behavior after annealing. As shown in Supplementary Fig. 13a, at a voltage of 0.6 V, the current abruptly increases, indicating the formation of a filament. The ions responsible for filament formation are likely copper ions that are decoupled from their binding sites due to thermal activation. Interestingly, after cooling the device back to room temperature, applying a negative voltage of −1.2 V causes a sharp drop in current (Supplementary Fig. 13b), suggesting the erasing of the ionic conductive filaments. In contrast to the abrupt changes induced by conductive filaments, the resistive switching reported in our manuscript exhibits slow, gradual changes in the I–V curves, forming a clear contrast to the abrupt changes caused by conductive filaments.

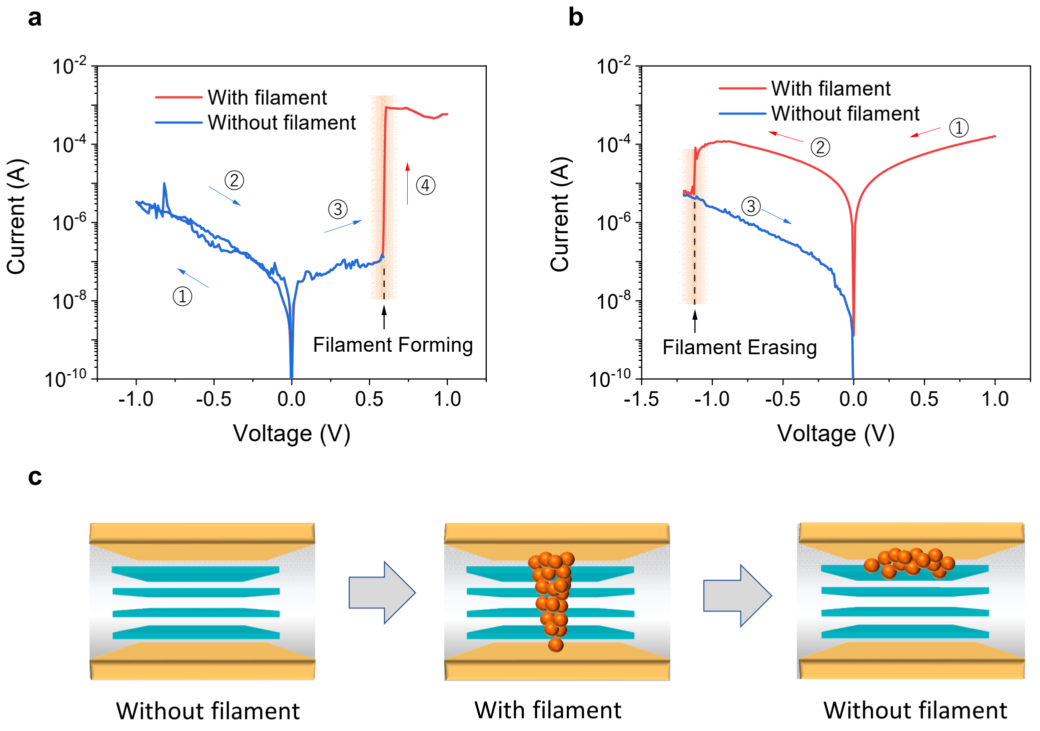

Supplementary Fig. 13 | Filamentary switching. a, Filament forming at high temperature. **b,** Filament erasing after cooling back. **c,** Schematic of filament forming and erasing.

Supplementary Note 5: Fitting with the Schottky-emission model

The current under the Schottky emission can be expressed as[1]:

$$I \propto T^2 \exp\left\{-\left[\frac{\Phi_B^0}{k_B T} - \frac{1}{k_B T}\left(\frac{q^3 V}{4\pi\varepsilon_0\varepsilon_r d}\right)^{\frac{1}{2}}\right]\right\}, \quad (1)$$

$$ln(I) \propto 2In(T) + \left(\frac{q}{k_B T}\right)\left(a\sqrt{V} - \Phi_B\right), \quad (2)$$

where Φ_B^0 is the Schottky potential barrier height without an external electric field, T is the Kelvin temperature, V is the applied voltage, ε_r is the relative dielectric constant, d is the ferroelectric flake thickness, and a is a constant equal to $\sqrt{\frac{q}{4\pi\varepsilon_0\varepsilon_r d}}$. The fitting result is shown in Supplementary Fig. 14, which indicates that the Schottky-emission model is only applicable at low voltages (0–0.53 V and 0–0.71 V for LRS and HRS, respectively).

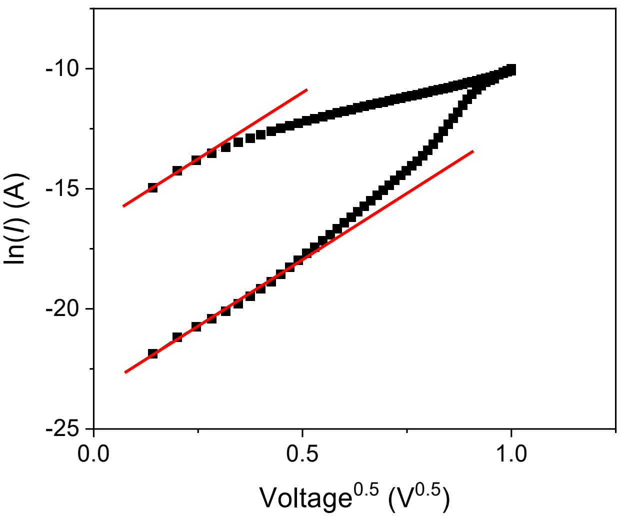

Supplementary Fig. 14 | Fitting result of the current via the Schottky-emission model. The relation between ln(I) and $V^{0.5}$ is linear only at a low voltage (0–0.53 V and 0–0.71 V for LRS and LRS, respectively), as marked by the red straight line.

Supplementary Note 6: Fitting with the Fowler–Nordheim tunneling model

The current density for the FN tunneling can be expressed as follows[2]:

$$J = \frac{q^2E^2}{16\pi^2\hbar\Phi}\exp\left(-\frac{4\sqrt{2m^*}(q\Phi)^{\frac{3}{2}}}{3q\hbar E}\right), \tag{3}$$

$$ln\left(\frac{J}{E^2}\right) = \left(\frac{1}{E}\right)\left(\frac{-4\sqrt{2m^*}(q\Phi)^{\frac{3}{2}}}{3q\hbar}\right) + ln\left(\frac{q^2}{16\pi^2\hbar\Phi}\right), \tag{4}$$

where Φ is the potential barrier height without an external electric field, m^* is the electron's effective mass, E is the electric field, and ħ is the reduced Planck constant. The ln($I\ V^{-2}$) versus V^{-1} plot is linearly fitted by $y = kx + b$, where $x = V^{-1}$, $y = \ln(I\ V^{-2})$ and the slopes (k) are extracted for the HRS and LRS at investigated temperatures as shown in Table S1. k is associated with the potential barrier height, which can be expressed as follows[1]:

$$k = \frac{-4\sqrt{2m^*}d(q\Phi)^{\frac{3}{2}}}{3q\hbar}, \tag{5}$$

$$b = ln\left(\frac{q^2l^2}{16\pi^2\hbar\Phi d^2}\right), \tag{6}$$

$$\Phi = \left(k \times \frac{3\hbar}{4\sqrt{2m^*q}}\right)^{\frac{2}{3}}. \tag{7}$$

Here, $m^* = 1.3m_0$, where m_0 is the rest mass of an electron. This corresponds to the fact that the effective mass is larger in the OOP direction due to the weak vdW interactions[5]. l and d are the width and the thickness of the ferroelectric layer, respectively. The data fitting leads to extracted electron-barrier heights of 0.138 eV and 0.08 eV for the HRS and LRS, respectively. In contrast to the Schottky-emission model, the fitting with FN-tunneling model is found to be applicable at a high voltage of 0.45–1 V.

Table S1: fitting parameters for F–N tunneling at investigated temperatures.

Temperature (°C)	k (HRS) (V)	k (LRS) (V)	b (HRS) (A V^{-2})	b (LRS) (A V^{-2})
27	−4.654	−2.677	−10.581	−12.558
47	−4.487	−2.522	−10.166	−12.131
67	−4.559	−2.494	−9.508	−11.573
87	−4.232	−2.204	−9.325	−11.353
97	−3.900	−1.006	−8.883	−11.777
107	−3.023	−0.338	−9.167	−11.852

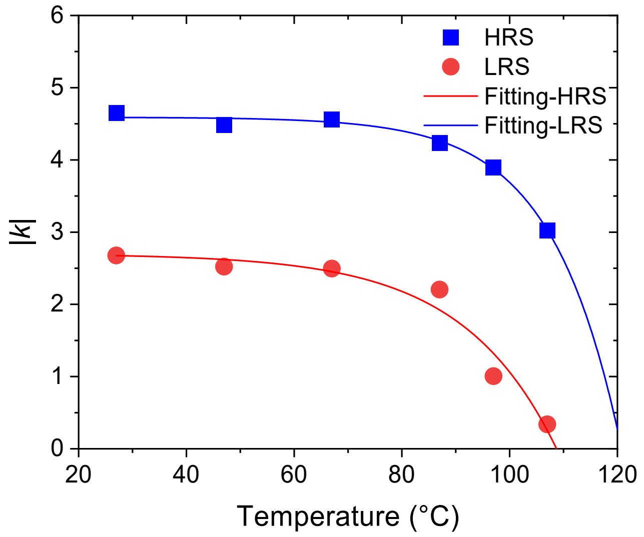

Supplementary Fig. 15 | Temperature dependence of fitting parameters for F–N tunneling.

The possible reasons for the temperature dependence are as follows. (1) Barrier height variation arising from the temperature-induced statistical carrier distribution; (2) Alterations in the band gap induced by thermal fluctuations; (3) Phonon-assisted tunneling. (4) The contribution of current from thermionic emission.

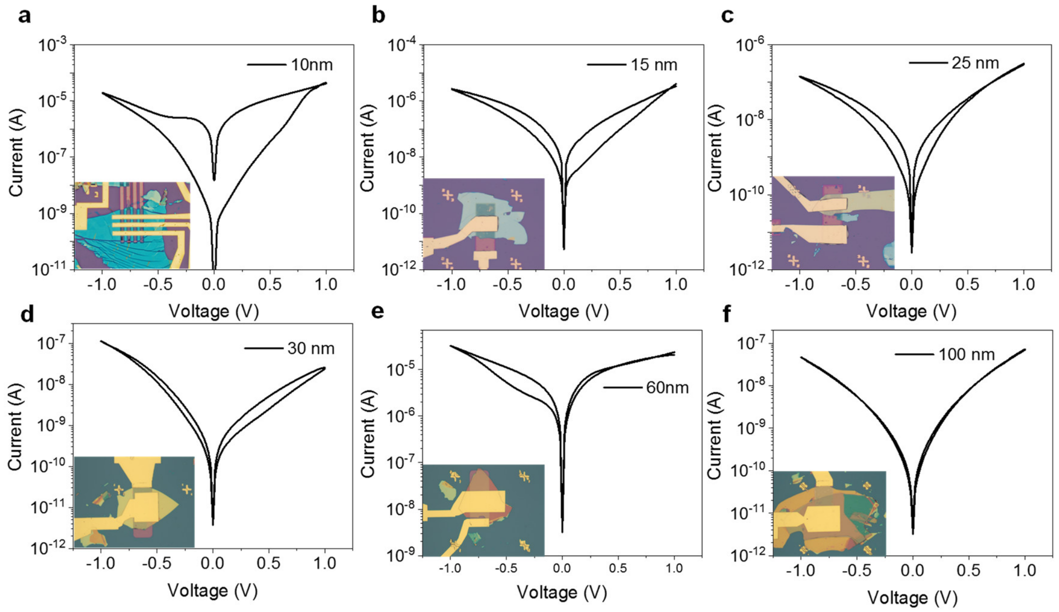

Supplementary Fig. 16 | *I*–*V* characteristics for samples with varying thickness. a, 10 nm. **b,** 15 nm. **c,** 25 nm. **d,** 30 nm. **e,** 60 nm. **f,** 100 nm.

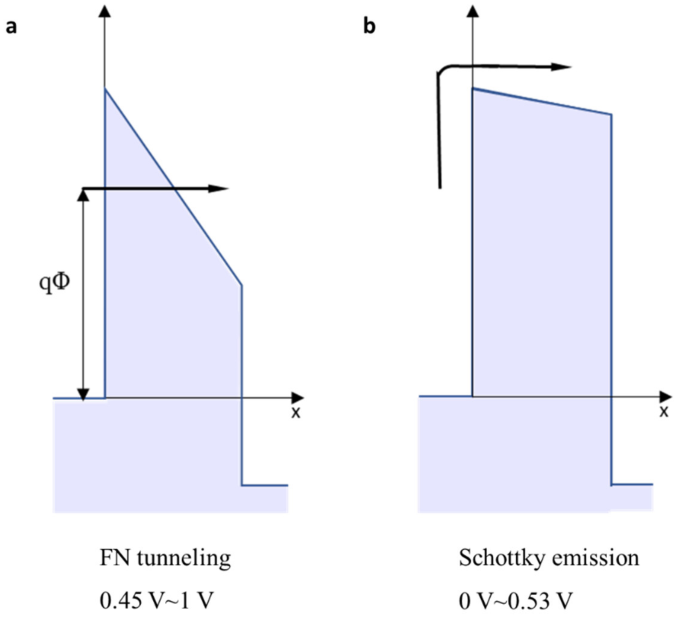

Supplementary Fig. 17 | Schematics of the energy barriers and electron-conduction processes. **a,** FN tunneling at high voltages. **b,** Schottky emission at low voltages. The barrier thickness decreases for FN tunneling at high voltages because of the strong external electric field. When the voltage is low, electrons can only pass through the barrier via thermionic emission.

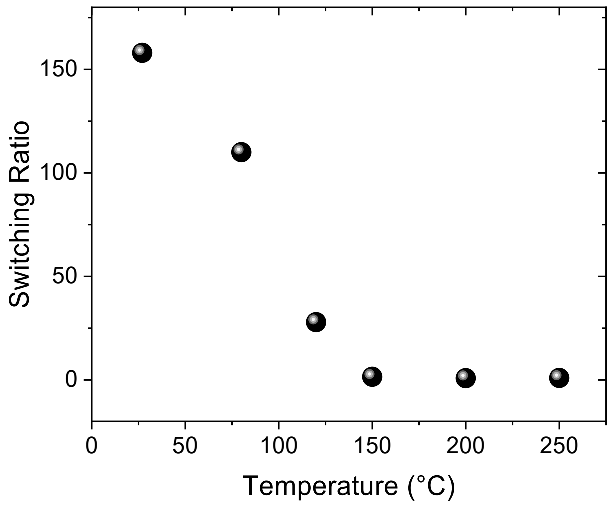

Supplementary Fig. 18 | Temperature dependence of the switching ratio of the memristor.

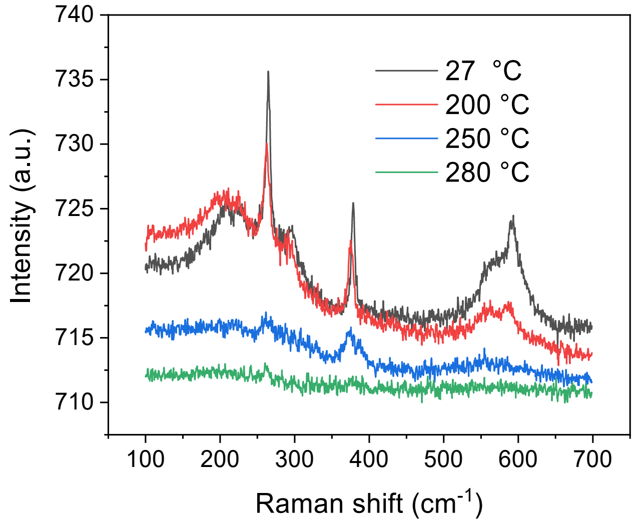

Supplementary Fig. 19 | Temperature-dependent Raman measurement on a 10-nm $CuCrP_2S_6$ flake.

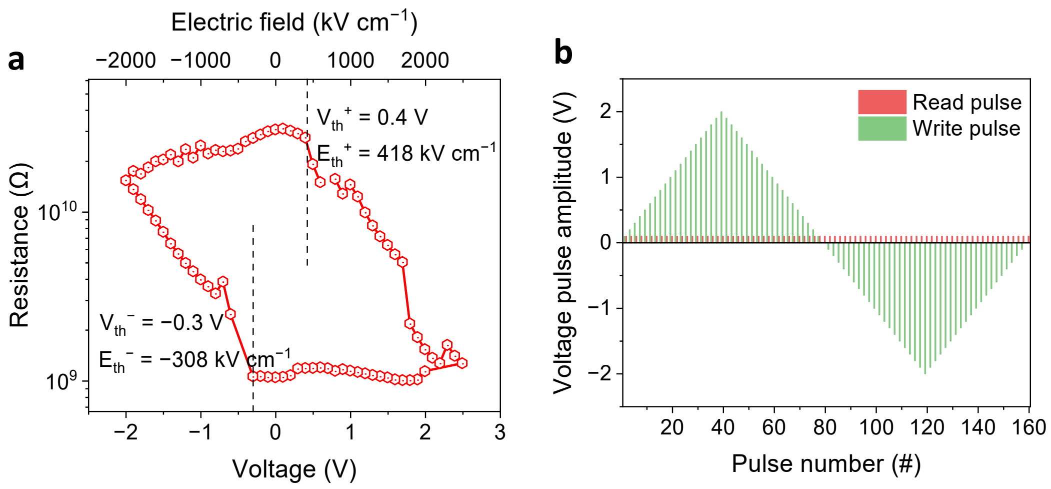

Supplementary Fig. 20 | Pulse test of resistance switching. **a,** Resistance hysteresis loop of $CuCrP_2S_6$ ferroelectric memristor displaying clear voltage thresholds. **b,** Voltage pulse train used for obtaining resistance hysteresis loop.

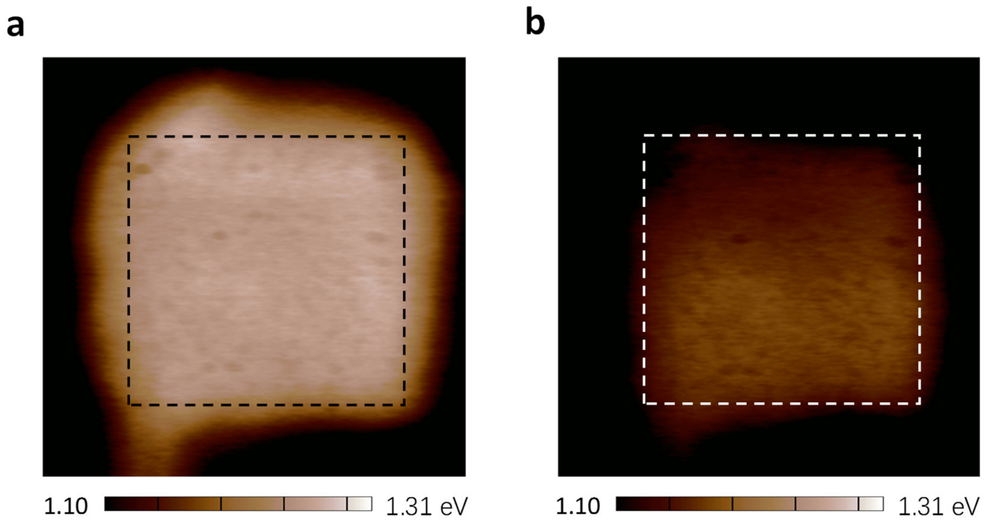

Supplementary Fig. 21 | Retention property for surface potential. a, surface potential image acquired with 5-minute delay after poled with the PFM tip. **b**, image acquired with 1-week delay. Poled regions are marked by dashed lines.

References

1. Perkins CK*, et al.* Demonstration of fowler-nordheim tunneling in simple solution-processed thin films. *ACS Appl. Mater. Interfaces* **10**, 36082-36087 (2018).
2. Yan F, Xing GZ, Li L. Low temperature dependent ferroelectric resistive switching in epitaxial $BiFeO_3$ films. *Appl. Phys. Lett.* **104**, 132904 (2014).
